# The Buried Bumper Syndrome: External Bumper Extraction after Radial Mini Incisions and Replacement through an Adjacent Tract

**DOI:** 10.1155/2016/5379291

**Published:** 2016-11-14

**Authors:** M. A. Benatta

**Affiliations:** ^1^Gastroenterology Department, Military Universitary Hospital, Oran, Algeria; ^2^Gastroenterology Department, Military Universitary Hospital, Constantine, Algeria; ^3^Digestive Endoscopy Unit, Central Hospital of Army, Algiers, Algeria

## Abstract

Although considered as a safe method to provide long-term nutritional support, percutaneous endoscopic gastrostomy (PEG) may be complicated by a buried bumper syndrome (BBS), a life-threatening condition. Removal of the PEG tube with its buried bumper and reinsertion of a new PEG tube is often necessary. Since its description in 1988, less than 50 cases of BBS managed by external extraction of the buried bumper have been reported. We report a case of buried bumper that was removed by external traction without the need for endoscopic or laparoscopic treatment but with the need of two radial millimeter skin incisions after abdominal CT study and finally immediate PEG replacement but through an adjacent site.

## 1. Introduction

Although considered as a safe method to provide long-term nutritional support, percutaneous endoscopic gastrostomy (PEG) may be complicated by a buried bumper syndrome (BBS). BBS occurs in approximately 0.3–2.4% of the patients [1] with a median length of time for the development of buried bumper syndrome of 18 months [2]. The mechanism involves excessive traction for an extended period leading to ischemia and ulceration of the mucosa between the internal and external bumpers. BBS is a life-threatening complication in which the internal bumper migrates from the gastric lumen and lodges anywhere along the gastrostomy tract. Removal of the PEG tube with its buried bumper and reinsertion of a new tube is often necessary. Although endoscopic methods such as the needle knife technique and the push-pull T-technique have been described, most buried inner bumpers are removed surgically under local anaesthesia or after laparotomy. These approaches can be associated with pain, bleeding, wound infection, or a gastrocutaneous fistula. Since its description in 1988, less than 50 cases of BBS managed by external extraction have been reported. We report a case of buried internal bumpers that was removed by external traction without the need for endoscopic intervention but extracted two radial millimeter skin incisions.

## 2. Case Presentation

A 20-year-old man was referred at five months later to PEG insertion for dysphagia as neurologic sequelae of cervical trauma. The PEG was inserted using the “pull” technique without any complication and the internal bumper position was confirmed at endoscopic “second look.” A PEG with a soft silicone, externally removable internal bumper was used. Intermittent bolus feeding via the PEG was commenced on the same day. At 5 months, the patient experienced abdominal pain and the nurses noticed slow flow during PEG feeding. Physical examination of the patient revealed a localized tenderness and erythematous circumferential induration about 2 cm surrounding the stomal site was seen. The internal bumper was palpated in the abdominal wall. With the exception of leukocytosis (14 × 10^3^/*μ*L), laboratory studies were unremarkable. The buried bumper syndrome was shown endoscopically, with findings of nonvisualization of the internal bumper, but a depressed and puckered area of the anterior gastric wall was seen, consistent with the embedded bumper into the gastric wall. Gentle flushing of the PEG tube externally was met with resistance but was seen entering the stomach through the puckered mucosal area without external leakage around the PEG site.

**Figure 1:**
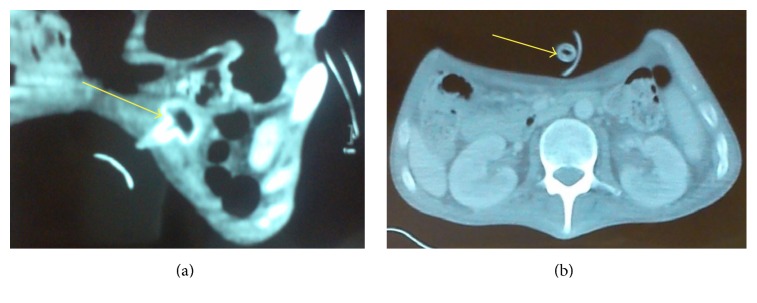
Abdominal computed tomography revealed the internal bumper buried in the gastric wall (yellow arrows) but without subcutaneous tissue or intra-abdominal infection.

To determine the exact location of the PEG internal bumper as well as any complications resulting from its migration, a computed tomography (CT) of the abdomen was performed and demonstrated the internal bumper in the gastric wall but without subcutaneous tissue or intra-abdominal infection (Figures 1(a) and 1(b)). Based on this study, under sedation, with gentle external traction, the internal bumper was easily extracted after two radial millimeter incisions of the abdominal wall. To insert a new PEG, during the same procedure, the guidewire externally introduced failed to pass into the gastric cavity through the original tract. Therefore, the new PEG was inserted using the usual “pull” technique but via an adjacent gastric tract. An endoscopic “second look” confirmed that the internal bumper was sitting on the adjacent entry site. The patient was started on broad spectrum antibiotics and care was taken to avoid excessive pressure between the outer and internal bumpers. As expected, there was no leakage through the original tract because this one was completely obstructed. The site continued to improve and the course was uneventful 9 months later.

## 3. Discussion

The mechanism of BBS involves excessive traction leading to ischemia and ulceration of the mucosa between the internal and external bumpers. Risk factors for the development of BBS include excessive tightening of the external bumper against the abdominal wall, inadequate patient care, and obesity [3]. Tubes with a small inner bumper, sharp tapered flange, and hard plastic composition may increase the risk of this complication [4]. Difficulty to infuse feeding solution through the tube due to complete or partial mucosal overgrowth burying the internal bumper and/or increased leakage around the PEG tube site leads to the diagnosis. A buried bumper can result in perforation of the stomach, abdominal wall abscess, peritonitis, necrotizing fasciitis, and death. Fortunately, in our patient, CT of the abdomen demonstrated the internal bumper in the gastric wall but without subcutaneous tissue or intra-abdominal infection (Figures 1(a) and 1(b)). The internal bumper removal may be obtained in cases of partial or superficial burial, with the “needle knife” [5], and in cases of a deep impaction with minimally invasive surgery. Removing internal bumper in BBS by external traction has been reported to be successful [1, 2, 6–11]. However, this may result in tearing at the PEG site but the introducing of collapsible internal bumpers made of softer material allows a safer extraction without incision. A new PEG tube was immediately reinserted through a tract adjacent to the original. Using a different site to replace a new PEG tube may be preferred because of the peristomal infection. This method can be used if the internal bumper is demonstrated to be embedded in the superficial abdominal wall by abdominal CT. BBS prevention includes an additional 1.5 cm between the external bumper of the PEG tube and the skin, cleaning and monitoring of the external PEG site, pushing in and rotating of the tube prior to repositioning of the external bumper, avoidance of unnecessary external tube traction, and monitoring the external length of the tube [7].

## 4. Conclusion

BBS is rare and its early diagnosis is necessary because it is a potentially life-threatening complication. Treatment included safely removing of the buried bumper by external traction after two radial millimeter skin incisions allowed by abdominal CT study and its immediate replacement with a pull-type PEG.
